# Tuning the electronic band structure of PCBM by electron irradiation

**DOI:** 10.1186/1556-276X-6-545

**Published:** 2011-10-04

**Authors:** Seung Hwa Yoo, Jong Min Kum, Sung Oh Cho

**Affiliations:** 1 Department of Nuclear and Quantum Engineering, Korea Advanced Institute of Science and Technology, Daejeon 305-701, South Korea

**Keywords:** tunable band structure, HOMO, LUMO, organic semiconductor, PCBM, electron irradiation

## Abstract

Tuning the electronic band structures such as band-edge position and bandgap of organic semiconductors is crucial to maximize the performance of organic photovoltaic devices. We present a simple yet effective electron irradiation approach to tune the band structure of [6, 6]-phenyl-C61-butyric acid methyl ester (PCBM) that is the most widely used organic acceptor material. We have found that the lowest unoccupied molecular orbital (LUMO) level of PCBM up-shifts toward the vacuum energy level, while the highest occupied molecular orbital (HOMO) level down-shifts when PCBM is electron-irradiated. The shift of the HOMO and the LUMO levels increases as the irradiated electron fluence increases. Accordingly, the band-edge position and the bandgap of PCBM can be controlled by adjusting the electron fluence. Characterization of electron-irradiated PCBM reveals that the variation of the band structure is attributed to the molecular structural change of PCBM by electron irradiation.

## Introduction

Organic semiconductors such as small molecules [1,2] and conjugated polymers [3,4] are widely used in organic photovoltaic cells [4-6], dye-sensitized solar cells [2,7], organic field-effect transistors [8-10], and organic light-emitting diodes [3,11]. In particular, [6,6]-phenyl-C61-butyric acid methyl ester (PCBM) is a small molecule that is most widely used as an electron acceptor in organic photovoltaic (OPV) cells [1]. To improve the power conversion efficiency of OPV cells, open-circuit voltage (*V*_oc_) of the cells should be increased. The upper limit of the *V*_oc _is determined by the energy difference between the highest occupied molecular orbital (HOMO) level of the electron donor and the lowest unoccupied molecular orbital (LUMO) level of the electron acceptor [12]. Thus, several efforts have been made to increase the LUMO level of PCBM by chemical approach, for instance, placing electron-donating and electron-withdrawing substituents on the phenyl ring or synthesizing bisadduct analogue of PCBM [12-14]. However, these approaches generally require complicated synthetic procedures and result in a low yield of the products [13]. As an alternative, radiation chemistry can be a good strategy to modify the chemical structures of particularly organic materials [15-18]. As a result of the chemical structural modification, the optical properties of the organic materials can be changed [19-21]. Here, we present a simple and novel approach to tune the HOMO and LUMO levels of PCBM based on electron irradiation. Only by irradiating an electron beam onto PCBM, the bandgap as well as the HOMO and LUMO levels of PCBM can be changed, and furthermore the electronic band structures of PCBM can be controlled by adjusting the electron fluence.

## Results and discussion

**Figure 1:**
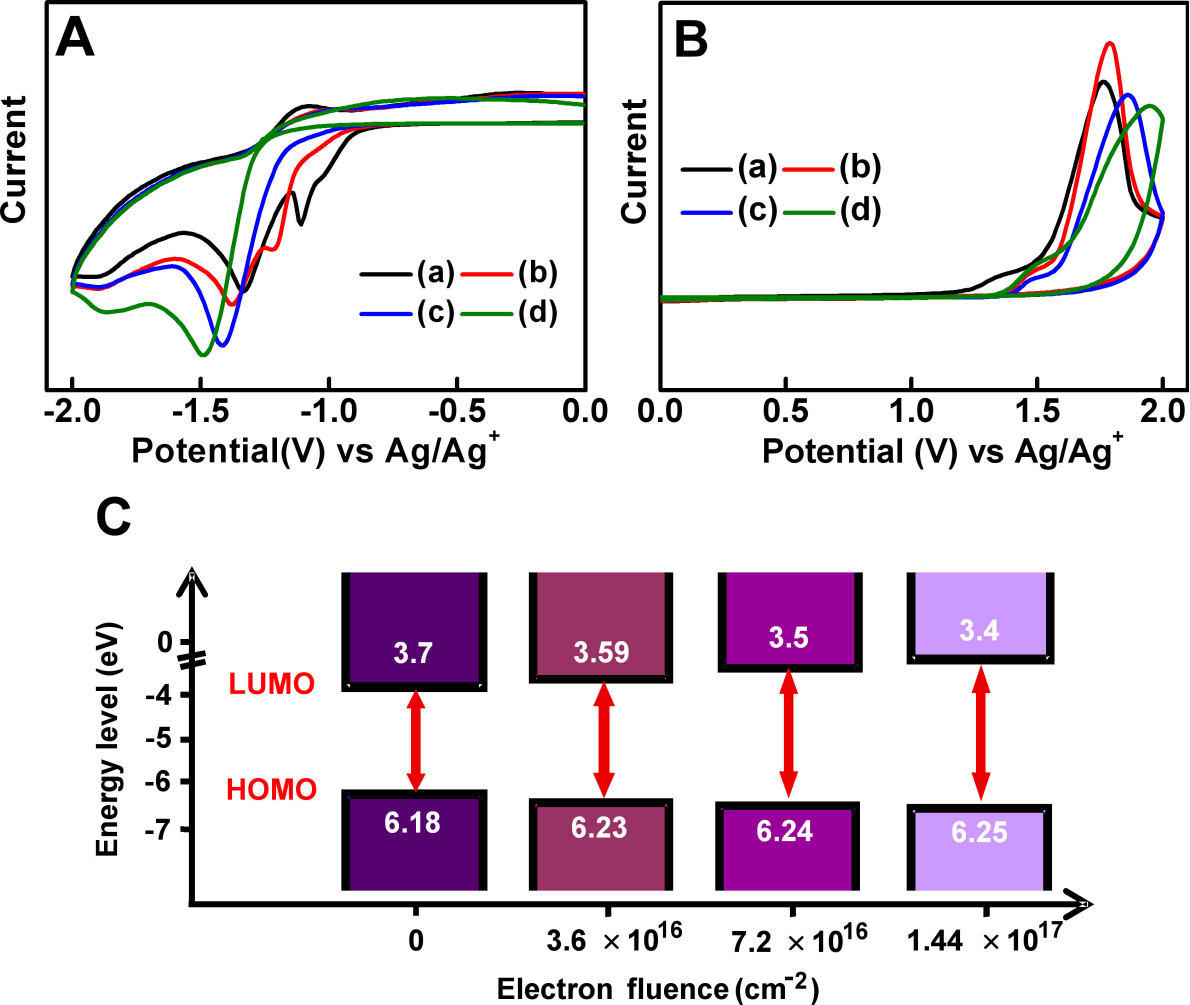
**Reduction and oxidation properties of electron-irradiated PCBM thin films, pristine PCBM measured by cyclic voltammetry**. Cyclic voltammogram of cathodic scan (**A**) and anodic scan (**B**) of PCBM film after electron irradiation at different fluences of (a) 0, (b) 3.6 × 10^16 ^cm^-2^, (c) 7.2 × 10^16 ^cm^-2^, and (d) 1.44 × 10^17 ^cm^-2^. (**C**) HOMO and LUMO levels of PCBM changed by electron irradiation as a function of electron fluence.

Figure 1 shows the reduction and oxidation properties of electron-irradiated PCBM thin films as well as pristine PCBM measured by cyclic voltammetry (CV). Pristine PCBM exhibits three reduction peaks (-1.11 V, -1.33 V, and -1.92 V vs. Ag/Ag^+^) and one oxidation peak (+1.76 V vs. Ag/Ag^+^). Electron irradiation led to a negative shift of the reduction peaks and a positive shift of the oxidation peak. Interestingly, only two reduction peaks appeared for the electron-irradiated PCBM at fluences higher than 7.2 × 10^16 ^cm^-2^. The first reduction peak of pristine PCBM was located at -1.11 V vs. Ag/Ag^+^; however, the peak was negatively shifted to -1.22 V, -1.41 V, and -1.49 V as the electron fluence was increased to 3.6 × 10^16^, 7.2 × 10^16^, and 1.44 × 10^17 ^cm^-2^, respectively. On the contrary, the oxidation peak was positively shifted from the pristine value of 1.76 to 1.79, 1.86, and 1.94 V with increasing the electron fluence to 3.6 × 10^16^, 7.2 ×, and 1.44 × 10^17^, respectively.

From the CV measurements, the LUMO and HOMO levels of PCBM were calculated using the following equation [22]:

(1) $$\text{LUMO}\;\;\left(\text{HOMO}\right)\;\;\left(\text{eV}\right)\;\text{ = }-\text{4}.\text{8}-\left(E_{\text{on}}-E_{1∕2}\left(\text{ferrocene}\right)\right)$$

where *E*_on _is the onset potential of the first reduction peak for LUMO or the onset potential of the oxidation peak for HOMO, and *E*_1/2 _(ferrocene) is the half-wave potential of a ferrocene redox reaction. The LUMO level up-shifts toward the vacuum energy level, while the HOMO level down-shifts with increasing the electron fluence (Figure 1C, the detailed values are shown in Table 1). The LUMO level of the irradiated PCBM was increased by 0.3 eV and the HOMO level was decreased by 0.07 eV compared to the respective values of the pristine PCBM when the electron fluence was 1.44 × 10^17 ^cm^-2^. Consequentially, the bandgap of electron-irradiated PCBM gradually increases with increasing the electron fluence: the bandgap increases from the pristine value of 2.48 to 2.85 eV at the electron fluence of 1.44 × 10^17 ^cm^-2^.

**Table 1:** HOMO and LUMO levels of PCBM changed by electron irradiation as a function of electron fluence

	E_p, red(1)_	*E*_on, red (1)_	LUMO	*E*_p, ox_	*E*_on, ox_	HOMO
PCBM	-1.11	-0.98	-3.70	1.76	1.50	-6.18
PCBM (3.6 × 10^16 ^cm^-2^)	-1.22	-1.09	-3.59	1.79	1.55	-6.23
PCBM (7.2 × 10^16 ^cm^-2^)	-1.41	-1.18	-3.50	1.86	1.56	-6.24
PCBM (1.44 × 10^17 ^cm^-2^)	-1.49	-1.28	-3.40	1.94	1.57	-6.25

The first reduction and oxidation peak potentials (voltage vs. Ag/Ag^+^), onset potentials, and LUMO and HOMO level (electron volts) of electron-irradiated PCBM at different fluences.

**Figure 2:**
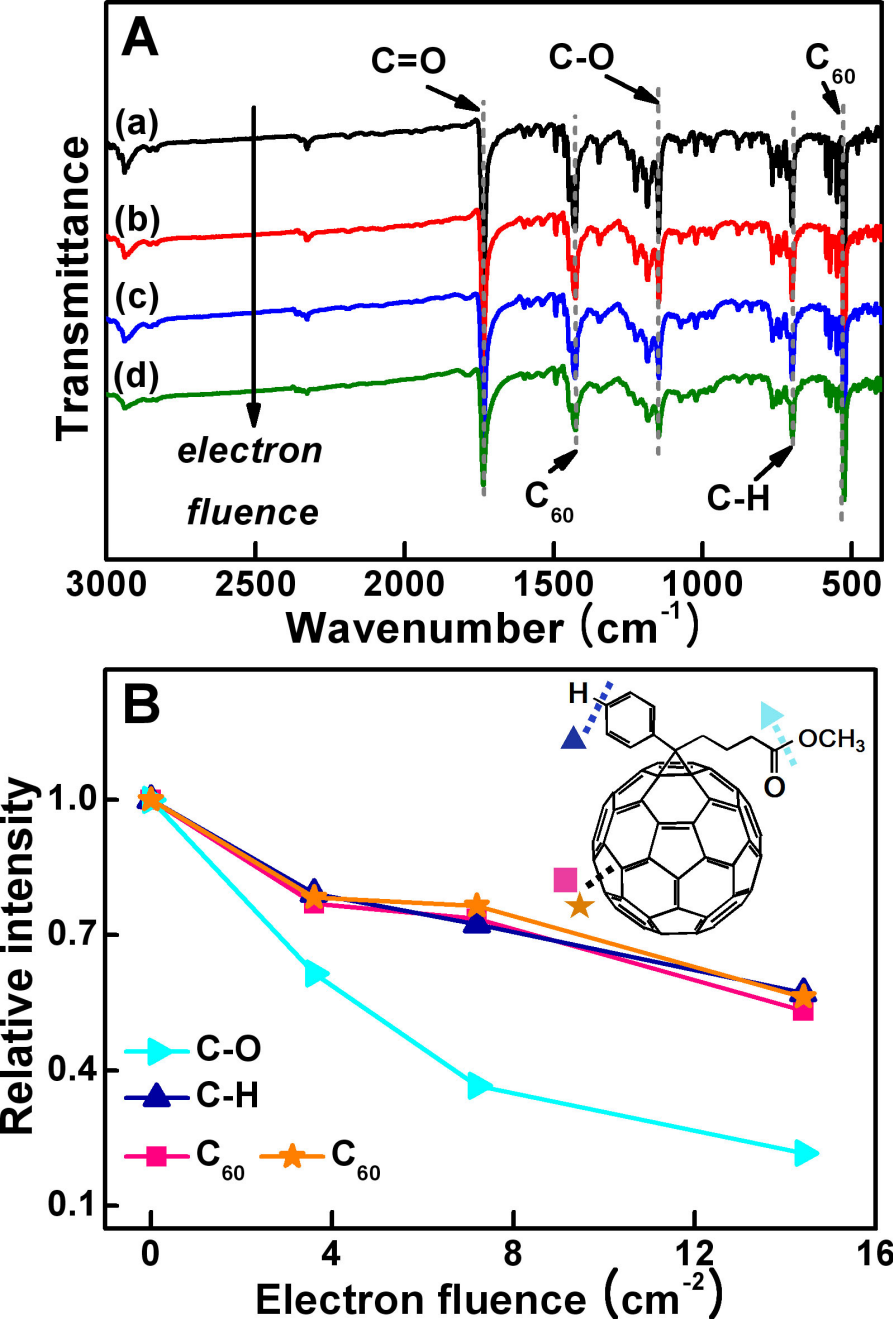
**FTIR spectra**. (**A**) FTIR spectrum of electron-irradiated PCBM at electron fluence of (a) 0, (b) 3.6 × 10^16 ^cm^-2^, (c) 7.2 × 10^16 ^cm^-2^, and (d) 1.44 × 10^17 ^cm^-2 ^including assignment of several vibration modes with its peak position. (**B**) Relative intensity of vibration modes in the butyric acid methyl ester side chain and phenyl ring of electron-irradiated PCBM.

**Figure 3:**
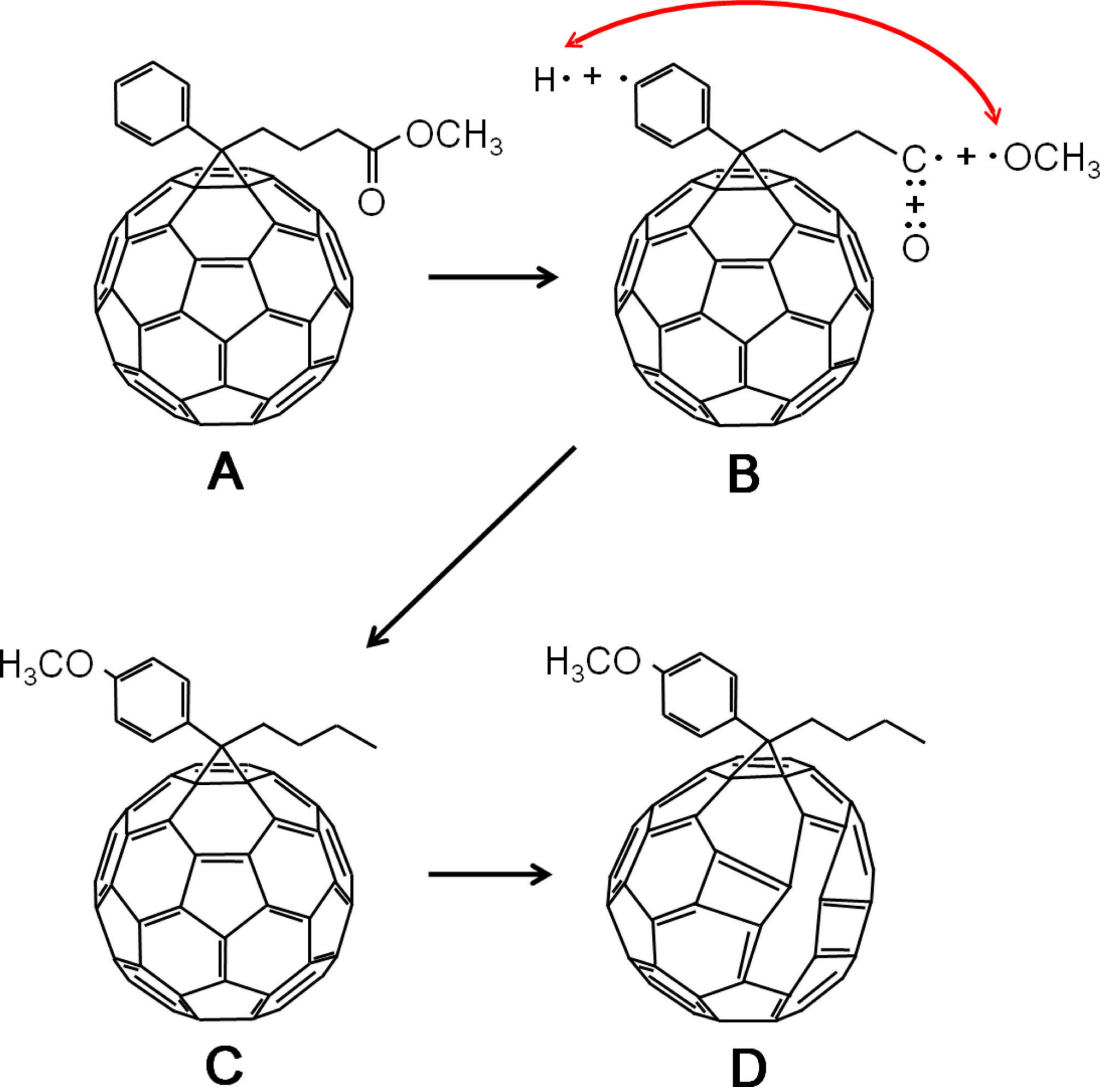
**Schematic illustration of the modification in the molecular structure of PCBM by electron irradiation**. (**A**) Molecular structure of pristine PCBM. (**B**) Formation of methoxy radical and phenyl radical by electron irradiation. (**C**) Formation of new methoxy-substituted phenyl ring by the combination of the methoxy radical and the phenyl radical. (**D**) Destruction of the C_60 _cage by high-dose electron irradiation.

**Figure 4:**
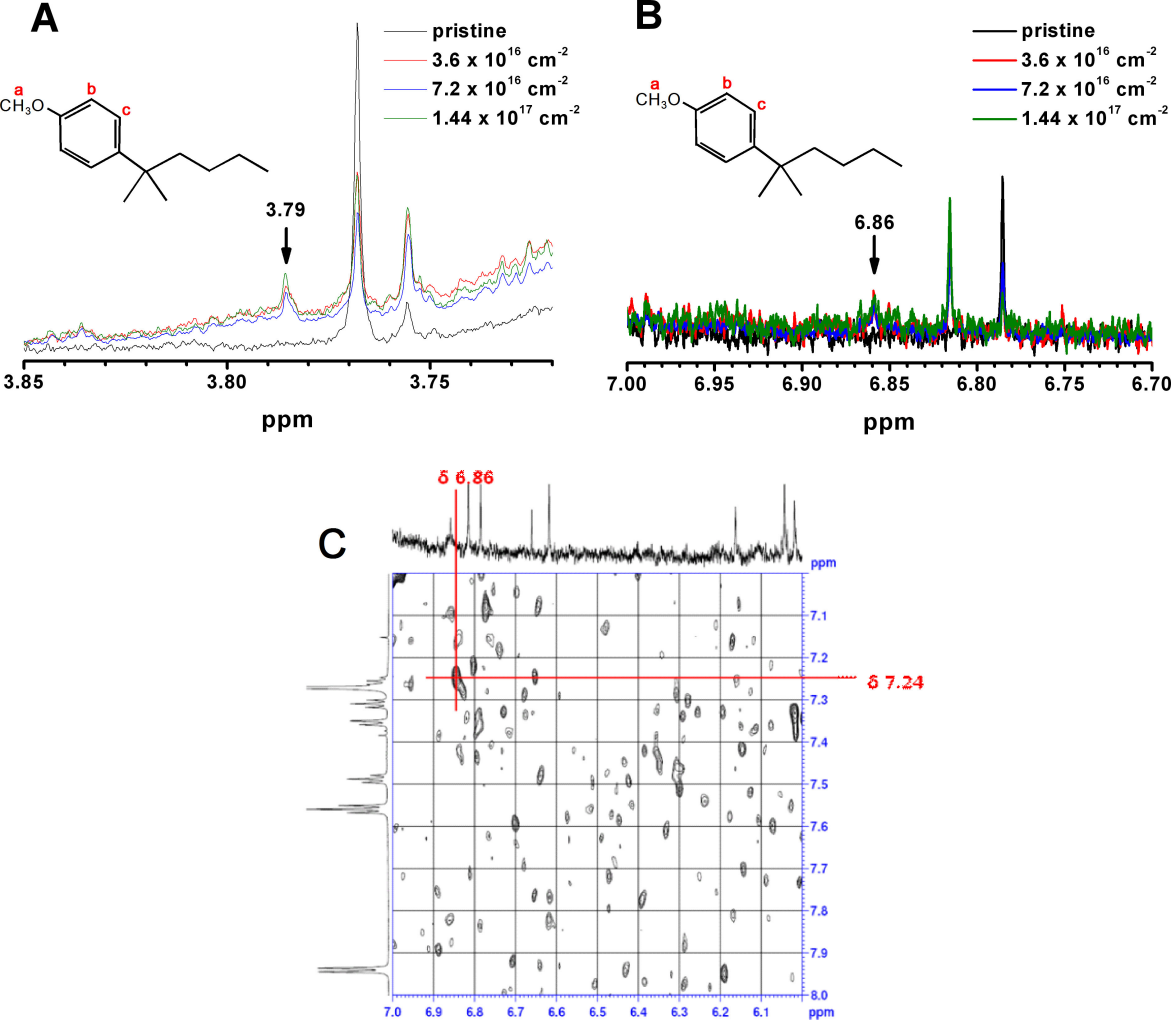
**1H NMR spectra of the electron-irradiated PCBM at different fluences**. (**A**) a new signal at 3.79 ppm develops, which corresponds to the hydrogen *a *of the methoxy group attached on the para position of phenyl ring in the PCBM. (**B**) Another new signal at 6.86 ppm corresponds to the hydrogen *b *on the meta position of phenyl ring in the PCBM. (**C**) The hydrogen *c *on the ortho position of phenyl ring is displayed in the 2D H-H COSY, which is positioned at 7.24 ppm.

To investigate the origin of the change in the band structure of electron-irradiated PCBM, the molecular structure of electron-irradiated PCBM was characterized by ^1^H nuclear magnetic resonance (NMR), Fourier transform infrared (FTIR), and Raman spectroscopy. PCBM is a fullerene derivative of which molecular structure comprises a side chain of butyric acid methyl ester and a phenyl ring attached on a C_60 _cage [1], FTIR spectra show that the intensities of all the peaks decreased with increasing the electron fluence (Figure 2A), suggesting that the molecular bonds of PCBM are gradually decomposed by electron irradiation. Analysis on the peak intensity variation reveals the C-O bond of the butyric acid methyl ester is most rapidly decomposed (Figure 2B). This indicates that the C-O bond can be easily detached from the side chain, forming methoxy radicals (Figure 3B). In addition, one of the C-H bonds in the phenyl ring was also broken by electron irradiation, producing phenyl radicals (Figure 3B). These two radicals produced by radiolysis make bonds with each other to form methoxy-substituted phenyl ring on PCBM (Figure 3C). The presence of methoxy-substituted phenyl ring is verified by ^1^H NMR spectra. First, a new signal at 3.79 ppm appeared and this signal corresponds to the hydrogen *a *marked in Figure 4A, that is, the hydrogen of methoxy group attached on the para position of phenyl ring in electron-irradiated PCBM. In addition, another new signal at 6.86 ppm (Figure 4B) and 7.24 ppm (Figure 4C) emerged and these signals are attributed to the hydrogen *b *on the meta position and hydrogen *c *on the ortho position of methoxy-substituted phenyl ring, respectively. It has been reported that attaching methoxy group on phenyl ring up-shifts the LUMO level of PCBM [12,23]. Therefore, we propose that the up-shift in the LUMO level of electron-irradiated PCBM is partly attributed to the attachment of methoxy group on the phenyl ring of PCBM.

**Figure 5:**
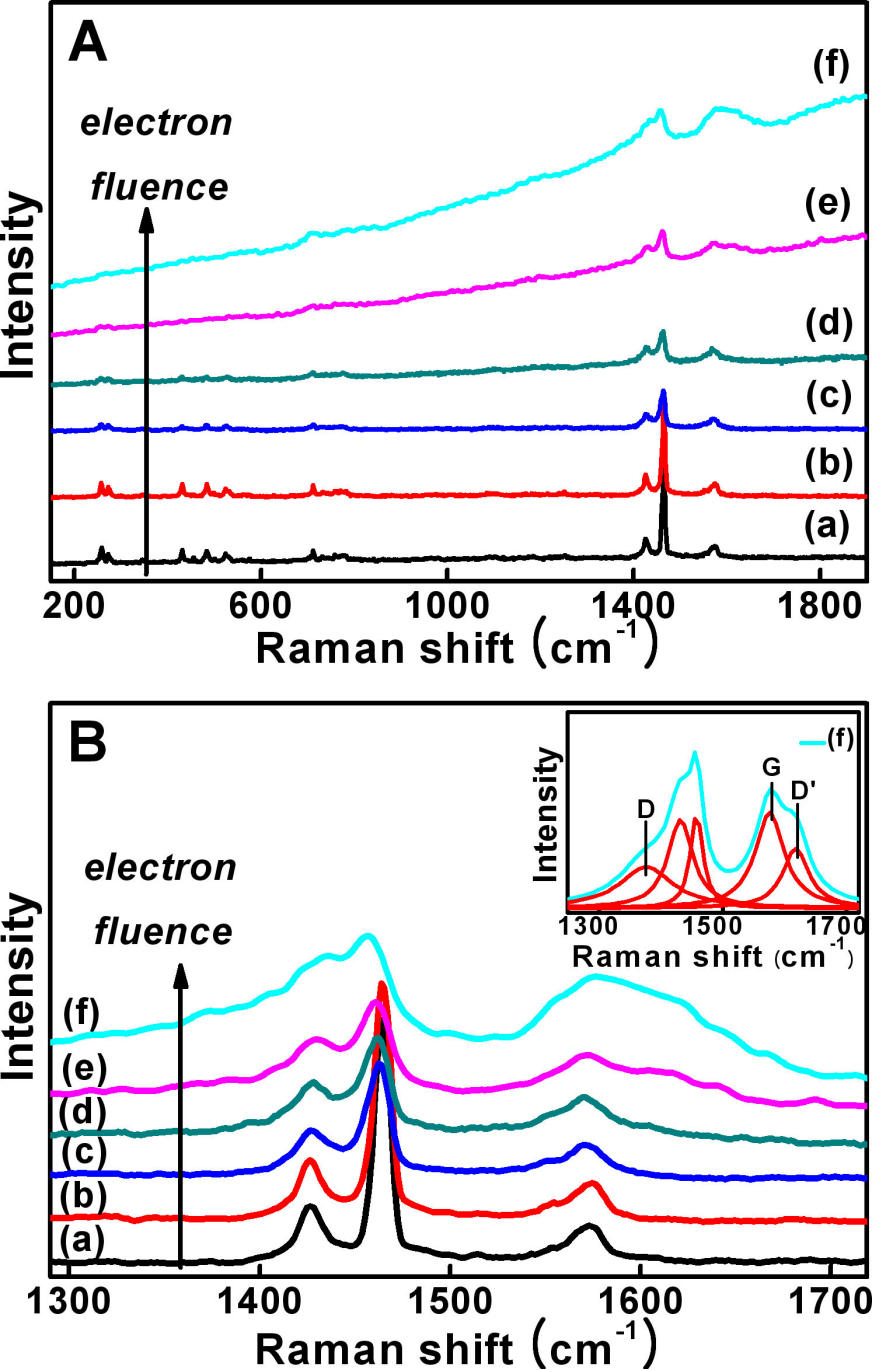
**Raman spectra**. (**A**) Raman spectra of electron-irradiated PCBM at different electron fluences of (a) 0, (b) 3.6 × 10^16 ^cm^-2^, (c) 7.2 × 10^16 ^cm^-2^, and (d) 1.44 × 10^17 ^cm^-2^, (e) 2.88 × 10^17 ^cm^-2^, and (f) 3.96 × 10^17 ^cm^-2^. (**B**) Raman spectra of 1,300- to approximately 1,700-cm^-1 ^region with the subtraction of the linear Raman scattering background for peak analysis. The upper right inset shows the deconvoluting peaks in the 1,300- to approximately 1,700-cm^-1 ^spectra region of (f) in (B).

Along with the modification in the side chain of butyric acid methyl ester, C_60 _backbone in PCBM molecule was also deformed by electron irradiation. Figure 2B verifies this fact that the intensities of peaks associated with the C_60 _cage gradually decreases as the electron fluence increases. For more detailed interpretation of the modification in C_60 _cage, electron-irradiated PCBM was analyzed by Raman spectroscopy. PCBM shows ten Raman-active vibration modes, which originates from the icosahedral symmetry (*I_h_*) of C_60 _[24]. These vibration modes are sorely affected by change in the *I_h _*symmetry of C_60_, therefore, the structural change of C_60 _at different fluences of electron irradiation was analyzed by observing the variation of those vibration modes. At electron fluence of 3.6 × 10^16 ^cm^-2^, no significant change was observed in the Raman spectrum, which indicates the C_60 _cage was intact at low fluence of electron irradiation. However, the ten Raman-active vibration modes show progressive decrease in the peak intensity as the electron fluence increases. Decrease in the peak intensity and the peak broadening of the strongest peak at 1,464 cm^-1 ^(A_g_(2), "pentagonal pinch" mode) clearly proves that the cage was destroyed by further electron irradiation [25-28]. These facts lead to a conclusion that the C_60 _cage was degraded from its icosahedral symmetry by high dose of electron irradiation. After the electron irradiation at a high fluence of 2.88 × 10^17 ^cm^-2^, broad bands at 1,376, 1,618, and 1,578 cm^-1^, which respectively correspond to D-, D'-, and G-bands of graphite [29,30] appeared (inset of Figure 5B). In addition, the Raman spectra of the electron-irradiated PCBM exhibit photoluminescence background with a positive slope and the slope of the background increases with increasing the electron fluence (Figure 5A) [25,31]. These two results indicate that PCBM is transformed into hydrogenated amorphous carbon structure at high dose of electron irradiation (Figure 3D).

Consequently, from these analyses, we can conclude that the change in the band structure of electron-irradiated PCBM is attributed to the modification of the molecular structure of PCBM by electron irradiation (Figure 3). Formation of methoxy-substituted phenyl ring on PCBM up-shifts the LUMO level at low electron fluencies; further electron irradiation deforms the C_60 _cage and gradually converts it to hydrogenated amorphous carbon, resulting in the increase of the HOMO-LUMO gap. This also indicates that the band structure of PCBM can be tuned by adjusting the electron dose.

## Conclusions

We have found that the electronic band structure of PCBM is changed by electron irradiation. The LUMO level of PCBM gradually up-shifts toward the vacuum energy level, while the HOMO level slightly down-shifts against the vacuum energy level as the electron fluence increase. Consequently, the bandgap of PCBM can be controlled by adjusting the electron fluence. The variation of the band structure is attributed to the change in the molecular structure of PCBM by electron irradiation. The electron irradiation technique can also be used to control the electronic band structures of other organic semiconductors and thus this irradiation technique can provide a useful strategy to improve the performances of organic photovoltaic and organic optoelectronic devices.

## Methods

PCBM solution was prepared by dissolving PCBM (99.5% purity, Nano-C, Inc., Westwood, MA, USA) powder into chlorobenzene (≥ 99.5% purity, Sigma-Aldrich, St. Luois, Mo, USA). PCBM films were fabricated on glassy carbon electrodes by spin-coating a chlorobenzene solution containing 24 mM PCBM at 2,000 rpm for 60 s. The irradiation of an electron beam on PCBM films were carried out at room temperature and in vacuum lower than 2 × 10^-5 ^Torr. An electron beam was generated from a thermionic electron gun with electron energy of 50 keV and current density of the electron beam was 1.6 μA cm^-2 ^[16,32]. The electron fluence was varied by adjusting the irradiation time. PCBM films were irradiated by 1, 2, and 4 h, which corresponds to electron fluence of 3.6 × 10^16^, 7.2 × 10^16^, and 1.44 × 10^17 ^cm^-2^, respectively.

After electron irradiation of PCBM, the reduction and oxidation properties of PCBM were characterized by CV. The CV measurements were carried out using a three-electrode system consisting of the glassy carbon electrode as a working electrode, a platinum (Pt) wire as a counter electrode, and a Ag/Ag^+ ^electrode as a reference electrode in an acetonitrile solution of 0.1 M Bu_4_NPF_6_. Potentials were quoted with reference to the internal ferrocene standard (*E*_1/2 _= 0.120 V vs. Ag/Ag^+^) that was measured in the same electrolyte. The scan rate was 100 mV s^-1 ^for all measurements. The changes in molecular structure of PCBM due to electron irradiation were investigated by ^1^H NMR (Bruker Biospin AvanceII 900, Bruker, Billerica, MA, USA), FTIR and high-resolution dispersive Raman spectroscopy (Jasco FT/IR-4100 (JASCO, Easton, MD, USA) and LabRAM HR UV/Vis/NIR (HORIBA Jobin Yvon, Edison, NJ, USA), respectively).

## Competing interests

The authors declare that they have no competing interests.

## Authors' contributions

The work was carried out by collaboration between all authors. SOC initiated the idea of electron irradiation on PCBM. SHY performed the electron irradiation experiments and JMK carried out the FTIR, Raman, and NMR measurements of electron-irradiated PCBM. SOC and SHY analyzed the data and suggested the mechanism of band-tuning of electron-irradiated PCBM. All authors read and approved the final manuscript.
